# Estimation of Longitudinal Force and Sideslip Angle for Intelligent Four-Wheel Independent Drive Electric Vehicles by Observer Iteration and Information Fusion

**DOI:** 10.3390/s18041268

**Published:** 2018-04-20

**Authors:** Te Chen, Long Chen, Xing Xu, Yingfeng Cai, Haobin Jiang, Xiaoqiang Sun

**Affiliations:** 1 School of Automotive and Traffic Engineering, Jiangsu University, Zhenjiang 212013, China; ujschente@163.com (T.C.); xuxing@mail.ujs.edu.cn (X.X.); caicaixiao0304@126.com (Y.C.); jianghb@ujs.edu.cn (H.J.); sxq@ujs.edu.cn (X.S.); 2 Automotive Engineering Research Institute, Jiangsu University, Zhenjiang 212013, China

**Keywords:** distributed drive electric vehicle, state estimation, longitudinal force, sideslip angle, observer iteration

## Abstract

Exact estimation of longitudinal force and sideslip angle is important for lateral stability and path-following control of four-wheel independent driven electric vehicle. This paper presents an effective method for longitudinal force and sideslip angle estimation by observer iteration and information fusion for four-wheel independent drive electric vehicles. The electric driving wheel model is introduced into the vehicle modeling process and used for longitudinal force estimation, the longitudinal force reconstruction equation is obtained via model decoupling, the a Luenberger observer and high-order sliding mode observer are united for longitudinal force observer design, and the Kalman filter is applied to restrain the influence of noise. Via the estimated longitudinal force, an estimation strategy is then proposed based on observer iteration and information fusion, in which the Luenberger observer is applied to achieve the transcendental estimation utilizing less sensor measurements, the extended Kalman filter is used for a posteriori estimation with higher accuracy, and a fuzzy weight controller is used to enhance the adaptive ability of observer system. Simulations and experiments are carried out, and the effectiveness of proposed estimation method is verified.

## 1. Introduction

Electric vehicles (EVs), are a promising pattern of future transportation, which possess great advantages in fuel economy and emissions, and drawn lots of attention of both researchers and companies [1,2,3]. In particular, due to the accuracy and flexibility of torque control, four-wheel independent drive electric vehicles (4WID-EVs) have shown potential and effectiveness to improve vehicle stability [4,5,6]. The stable performance of vehicle motion control relies on precise and credible vehicle state measurements [7,8]. Recently, the study of intelligent automobiles and unmanned driving has aroused a great interest as an effective method with autonomous vehicle security and reduced mobility costs [9,10,11]. The traffic environment perception and estimation of important vehicle states is one of the keys to achieve vehicle path-following and stability control. Considering the fact that some vehicle states are hard and costly to measure, a model-based vehicle state estimator using low-cost sensors is essential to obtain the needed estimations.

In the past decade, there have been many studies in the field of longitudinal force and sideslip angle estimation. The algorithms utilized for observer design in prior papers include Kalman-filter-based methods [12,13,14,15,16], nonlinear-observer-based methods [17,18,19,20,21], optimal estimation methods [22,23,24,25,26], information fusion estimation methods [14,16,27,28,29,30], robust estimation methods [31,32], etc. Almost all the literature mentioned above focused on the enhancement of estimation performance, and some is also concerned with the reduction of estimation costs [22,30,32,33]. Baffet proposed an adaptive tire-force model with the consideration of road friction variations, and utilized the sliding-mode observer to estimate the tire-road forces [18]. Longitudinal force is a significant vehicle parameter, but in most existing studies, the longitudinal force observer is usually devised for traditional internal combustion engine vehicles, and longitudinal force estimation approaches for EVs, especially for 4WID-EVs, are still relatively hard to find. In some studies, the longitudinal forces are usually calculated by integrating the rotational dynamics differential equation of the driving wheels, but the noise is integrated at the same time, so the estimation accuracy is not guaranteed [34]. There exist some studies achieving the driving torque of in-wheel motors via multiplying the current of in-wheel motors by the gain which was calibrated by experimental data [2]. That is to say, the electric drive characteristics are not fully utilized for longitudinal force estimation in existing studies. The measurements by low-cost sensors, such as the current, rotational speed and bus voltage of in-wheel motors, can be used to estimate the longitudinal force via the dynamic characteristics of electric driving wheels. Few papers have taken this concept into account. The sideslip angle is pivotal for vehicle lateral stability and path-following control, and as the research has deepened, researchers tend to estimate the sideslip angle using more advanced filtering theories [14,16], by improving the reliability of estimation by means of observer iterations [16,29,30], or utilizing the information fusion estimation via the redundancy of measurements [22,23,27].

In this paper, a novel longitudinal force and sideslip angle estimation strategy with observer iteration and information fusion for 4WID-EVs is presented. The electric driving wheel model (EDWM) is introduced into the vehicle modeling process for longitudinal force estimation, the longitudinal force reconstruction equation is obtained by decoupling EDWM, and the nonlinear observer and high-order sliding mode observer [35,36,37] (HSMO) are combined to design the longitudinal force observer (LFO), and the Kalman filter (KF) is used to achieve an unbiased estimation of longitudinal force with the consideration of the in-system noise. This design provides a novel way of thinking about longitudinal force estimation in 4WID-EVs. In the sideslip angle estimation, the longitudinal forces estimated by LFOs are regarded as pseudo-measurements, the Luenberger observer (LO) is devised for transcendental estimation using less sensor measurements, the extended Kalman filter (EKF) is designed for a posteriori estimation with higher accuracy, the fuzzy weight which is changed with the variation of steering wheel angle and vehicle speed is applied to enhance the adaptive ability of vehicle state estimation.

The rest of this paper is organized as follows: the vehicle dynamic model is presented is Section 2. The LFO is designed in Section 3. The observer iteration-based sideslip angle estimator is described in Section 4. The simulation results are provided in Section 5. The experimental verifications are presented in Section 6, followed by our concluding remarks.

## 2. Vehicle Model

### 2.1. Vehicle Dynamics Model

**Figure 1:**
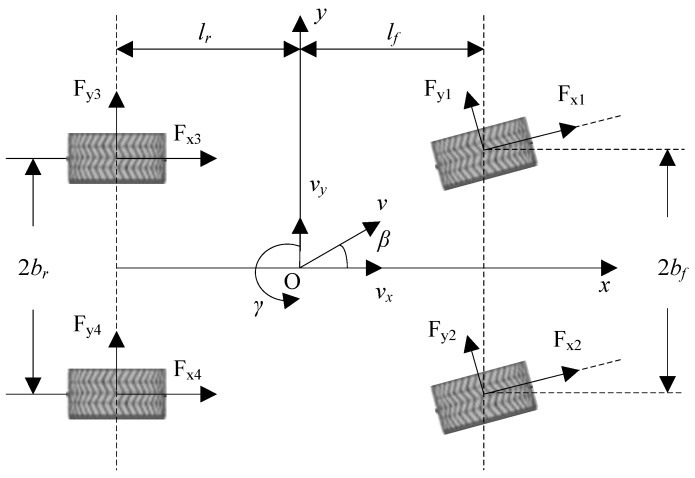
Vehicle model.

A schematic diagram of the four-wheel vehicle model in the longitudinal, lateral, and yaw directions is shown in Figure 1. The origin of the dynamic coordinate system *xoy* is fixed on the vehicle coinciding with the vehicle gravity center, the *x* axis is the longitudinal axis of the vehicle (the forward direction is positive), the *y* axis is the lateral axis of the vehicle (the right-to-left direction is positive). The pitch, roll, vertical motions and the suspension system of the vehicle are ignored. It is assumed that the mechanical properties of each tire are the same. The serial numbers 1, 2, 3, and 4 of the wheels respectively correspond to the front-left, the front-right, the rear-left and the rear-right wheel. The lateral velocity, yaw rate and sideslip dynamic equations of the four-wheel vehicle model can be expressed as:

(1) $$\overset{\cdot }{v_{y}}=-\gamma v_{x}+\frac{1}{m}[\left(F_{x1}+F_{x2}\right)\sin\delta +\left(F_{y1}+F_{y2}\right)\cos\delta +F_{y3}+F_{y4}]=-\gamma v_{x}+\frac{1}{m}\sum M_{y}$$

(2) $$\begin{array}{l} \overset{\cdot }{\gamma }=\frac{1}{I_{z}}[\left(F_{x1}+F_{x2}\right)l_{f}\sin\delta -(F_{y3}+F_{y4})l_{r}+\left(F_{y1}+F_{y2}\right)l_{f}\cos\delta +(F_{y1}-F_{y2})b_{f}\sin\delta \\ -(F_{x1}-F_{x2})b_{f}\cos\delta -(F_{x3}-F_{x4})b_{r}]=\frac{1}{I_{z}}\sum M_{z} \end{array}$$

(3) $$\begin{array}{l} \overset{\cdot }{\beta }=\frac{1}{mv}[-(F_{x1}+F_{x2})\sin(\beta -\delta )-(F_{x3}+F_{x4})\sin\beta +\\ \left(F_{y1}+F_{y2}\right)\cos(\delta -\beta )+(F_{y3}+F_{y4})cos\beta ]-\gamma =\frac{1}{mv}\sum M_{\beta }-\gamma \end{array}$$

where *v_y_* is the lateral vehicle speed at the center of gravity (COG), *γ* is the yaw rate which represents the angular velocity of vehicle at the COG, *β* is the sideslip of vehicle at the COG, *v* represents the velocity heading of vehicle at COG and $v=\sqrt{v_{x}^{2}+v_{y}^{2}}$, *v* is regarded to be approximately equal to *v_x_* only in (3) considering *v_y_* is relatively small, *m* represents the vehicle mass, *δ* is the steering angle of the front wheels, *I_z_* stands for the moment of inertia. *F_xj_* and *F_yj_* (*j* = 1, 2, 3, 4) are the longitudinal and lateral forces of the *j*th tire, respectively. *l_f_* and *l_r_* are the distances from vehicle gravity center to the front and rear axle, respectively. *b_f_* and *b_r_* are the half treads of the front wheels and rear wheels, respectively.

### 2.2. EDWM

**Figure 2:**
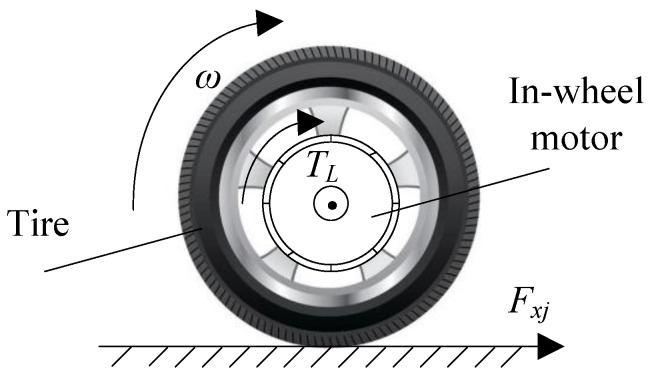
Vehicle model.

Each wheel of the 4WID-EV is driven by an in-wheel motor. The driving wheel, consisting of an in-wheel motor and tire, is an independent driving and informative unit. As shown in Figure 2, the concept of the EDMW is proposed in this study. The rotational dynamic equation of each wheel can be written as:

(4) $$J_{1}\overset{\cdot }{\omega_{j}}=T_{Lj}-F_{xj}r$$

where *ω_j_* is the wheel speed of the *j*th wheel, *J*_1_ is the wheel moment of inertia, *r* is the wheel effective rolling radius, *T_Lj_* is the load torque of in-wheel motor. The torque balance equation of the output shaft in in-wheel motor can be given by:

(5) $$J_{2}\overset{\cdot }{\omega_{j}}+b\omega_{j}=K_{t}i_{j}-T_{Lj}$$

where *J*_2_ is the rotational inertia of in-wheel motor rotor, *b* is the damping coefficient, *K_t_* is the motor torque constant, *i_j_* is the bus current. The dynamic voltage balance equation of equivalent circuit in in-wheel motor can be modeled as:

(6) $$u_{j}=Ri_{j}+L\overset{\cdot }{i_{j}}+K_{a}\omega_{j}$$

where *u_j_* is the bus voltage, *R* is the equivalent resistance of winding, *L* is the equivalent inductance of winding, *K_a_* is the inverse electromotive force coefficient.

### 2.3. Tire Model

The semi empirical magic formula of tire model is used to estimate the lateral tire force. The lateral tire force can be calculated as:

(7) $$F_{y}=D\sin\{C\arctan[B\alpha -E(B\alpha -\arctan(B\alpha ))]\}$$

where *B* is the stiffness factor, *C* is the curve shape factor, *D* is the peak factor, *E* is the curve curvature factor, *α* is the wheel side slip angle.

The tire model parameters like *B*, *C*, *D*, *E* are related to the tire vertical load. The vertical load of each tire can be calculated as:

(8) $$\left\{ \begin{array}{l} F_{z1}=l_{r}(\frac{mg}{2l}+\frac{ma_{y}h}{2b_{f}l})-\frac{ma_{x}h}{2l}\\ F_{z2}=l_{r}(\frac{mg}{2l}-\frac{ma_{y}h}{2b_{f}l})-\frac{ma_{x}h}{2l}\\ F_{z3}=l_{f}(\frac{mg}{2}+\frac{ma_{y}h}{2b_{r}l})+\frac{ma_{x}h}{2l}\\ F_{z4}=l_{f}(\frac{mg}{2}-\frac{ma_{y}h}{2b_{r}l})+\frac{ma_{x}h}{2l} \end{array}\right.$$

where *F_z_*_1_, *F_z_*_2_, *F_z_*_3_, and *F_z_*_4_ are the vertical load of corresponding tires, *h* is the height of the center of gravity, *g* is the acceleration of gravity. The side slip angle of each wheel can be obtained by:

(9) $$\left\{ \begin{array}{l} \alpha_{1}=\delta -\arctan\frac{v_{y}+l_{f}\gamma }{v_{x}+b_{f}\gamma /2}\\ \alpha_{2}=\delta -\arctan\frac{v_{y}+l_{f}\gamma }{v_{x}-b_{f}\gamma /2}\\ \alpha_{3}=-\arctan\frac{v_{y}-l_{r}\gamma }{v_{x}+b_{r}\gamma /2}\\ \alpha_{4}=-\arctan\frac{v_{y}-l_{r}\gamma }{v_{x}-b_{r}\gamma /2} \end{array}\right.$$

## 3. LFO Design 

The electromechanical coupling driving characteristics of 4WID-EVs provide a novel way of thinking in longitudinal force estimation. If we apply the current, speed and voltage of the EDWM, which can be measured by low-cost sensors, to the longitudinal force estimation, it is conducive to making full use of the advantages of 4WID-EVs and reducing the estimation costs.

By substituting Equations (4) into (5) and combining it with (6), we have:

(10) $$\left\{ \begin{array}{l} \overset{\cdot }{i_{j}}=-\frac{R}{L}i_{j}-\frac{K_{a}}{L}\omega_{j}+\frac{1}{L}u_{j}\\ \overset{\cdot }{\omega_{j}}=\frac{K_{t}}{J}i_{j}-\frac{b}{J}\omega_{j}-\frac{r}{J}F_{xj} \end{array}\right.$$

where *J* = *J*_1_ *+ J*_2_. The electric-driving wheel model (EDWM) is obtained as:

(11) $$\overset{\cdot }{x}=Ax+Bu+Dd+Ew$$

(12) y=Cx+Fv

where *x*, *u*, *d* and *y* is the state vector, the known input vector, and the unknown input vector and measurement vector, respectively. *w* and *v* are the uncorrelated zero mean white noise sequences. The known input and unknown input represents the voltage and longitudinal force, respectively, and, *x* = [*i_j_ ω_j_*]*^T^*, $A=\left[\begin{array}{cc} -\frac{R}{L} & -\frac{K_{a}}{L}\\ \frac{K_{t}}{J} & -\frac{b}{J} \end{array}\right]=\left[\begin{array}{c} A_{1}\\ A_{2} \end{array}\right]$, $B=\left[\begin{array}{c} \frac{1}{L}\\ 0 \end{array}\right]=\left[\begin{array}{c} B_{1}\\ 0 \end{array}\right]$, $D=\left[\begin{array}{c} 0\\ -\frac{r}{J} \end{array}\right]=\left[\begin{array}{c} 0\\ D_{2} \end{array}\right]$, $C=\left[\begin{array}{cc} 1 & 0\\ 0 & 1 \end{array}\right]=\left[\begin{array}{c} C_{1}\\ C_{2} \end{array}\right]$, $E=F=\left[\begin{array}{c} 1\\ 1 \end{array}\right]$.

Decoupling Equation (11), we have:

(13) $$\overset{\cdot }{x_{1}}=A_{1}x+B_{1}u+E_{1}w$$

(14) $$\overset{\cdot }{x_{2}}=A_{2}x+D_{2}d+E_{2}w$$

Using Equation (14), the analytic formula of longitudinal force is obtained as:

(15) $$d=D_{2}^{-1}(\overset{\cdot }{x_{2}}-A_{2}x-E_{2}w)$$

Assuming that *P =* $D_{2}^{-1}$, the reconstruction equation of longitudinal force is expressed as:

(16) $$\overset{\wedge }{d}=P(\overset{\cdot }{\overset{\wedge }{x_{2}}}-A_{2}\overset{\wedge }{x}-E_{2}w)$$

where $\overset{\wedge }{x}$ is the state estimation. A Luenberger observer (marked as LO1) is designed as:

(17) $$\overset{\cdot }{\overset{\wedge }{x}}=A\overset{\wedge }{x}+Bu+L(y-\overset{\wedge }{y})+D\overset{\wedge }{d}+Ew$$

where $\overset{\wedge }{y}=C\overset{\wedge }{x}$.

A new variable is constructed as:

(18) $$z=\overset{\wedge }{x}-DPy_{2}$$

It can be deduced as:

(19) $$\begin{array}{l} \overset{\cdot }{z}=\overset{\cdot }{\overset{\wedge }{x}}-DP\overset{\cdot }{y_{2}}=A\overset{\wedge }{x}+Bu+L(y-\overset{\wedge }{y})+Ew-DPC_{2}A\overset{\wedge }{x}-DPC_{2}Bu-DPC_{2}Ew\\ =(I-DPC_{2})A\overset{\wedge }{x}+(I-DPC_{2})Bu+(I-DPC_{2})Ew+L(y-\overset{\wedge }{y}) \end{array}$$

Defining that $T=I-DPC_{2}$, we get:

(20) $$\begin{array}{l} \overset{\cdot }{z}=TA\overset{\wedge }{x}+TBu+TEw+Ly+L\overset{\wedge }{y}=(TA-LC)\overset{\wedge }{x}+TBu+Ly+TEw\\ =(TA-LC)z+TBu+Ly+(TA-LC)DPy_{2}+TEw \end{array}$$

At this time, the unknown-input-free subsystem (13) can be represented as:

(21) $$\overset{\cdot }{z}=(TA-LC)z+TBu+Ly+(TA-LC)DPy_{2}+TEw$$

(22) $$\overset{\wedge }{x}=z+DPy_{2}$$

Considering the situation that there exists the noise in (21), the KF is designed to achieve the unbiased estimation of *z*. Then the estimation of $\overset{\wedge }{x}$ can be obtained by Equation (22).

With the known $\overset{\wedge }{x}$, if the differential of $\overset{\wedge }{x_{2}}$ is estimated, the longitudinal force can be obtained by Equation (16). Then according to Equation (14), we have:

(23) $$\overset{\cdot }{x_{2}}=A_{2}\overset{\wedge }{x}+D_{2}d+E_{2}w$$

Defining that:

(24) $$x_{3}=A_{2}\overset{\wedge }{x}+D_{2}d+Ew$$

It can be expressed as:

(25) $$\left\{ \begin{array}{l} \overset{\cdot }{x_{2}}=x_{3}\\ \overset{\cdot }{x_{3}}=\Phi (x,d,w) \end{array}\right.$$

Based on the super twisting algorithm, the HSMO is proposed as:

(26) $$\left\{ \begin{array}{l} \overset{\cdot }{\overset{\wedge }{x_{2}}}=\overset{\wedge }{x_{3}}-k_{x2}{\left|\overset{\wedge }{x_{2}}-x_{2}\right|}^{\frac{1}{2}}\mathrm{sgn}(\overset{\wedge }{x_{2}}-x_{2})\\ \overset{\cdot }{\overset{\wedge }{x_{3}}}=-k_{x3}\mathrm{sgn}(\overset{\wedge }{x_{3}}-k_{x2}{\left|\overset{\wedge }{x_{2}}-x_{2}\right|}^{\frac{1}{2}}\mathrm{sgn}(\overset{\wedge }{x_{2}}-x_{2})) \end{array}\right.$$

where $\overset{\wedge }{x_{2}}$ and $\overset{\wedge }{x_{3}}$ are the exact estimation of *x*_2_ and *x*_3_ in finite time, respectively. The coefficient *k_x_*_2_ and *k_x_*_3_ are the gains of HSMO and designed as follows:

(27) $$\left\{ \begin{array}{l} {\overset{\cdot }{k}}_{x2}=e_{2}{\left|e_{2}\right|}^{1/2}\mathrm{sgn}(e_{2})\\ {\overset{\cdot }{k}}_{x3}=e_{3}\mathrm{sgn}(e_{3}) \end{array}\right.$$

**Figure 3:**
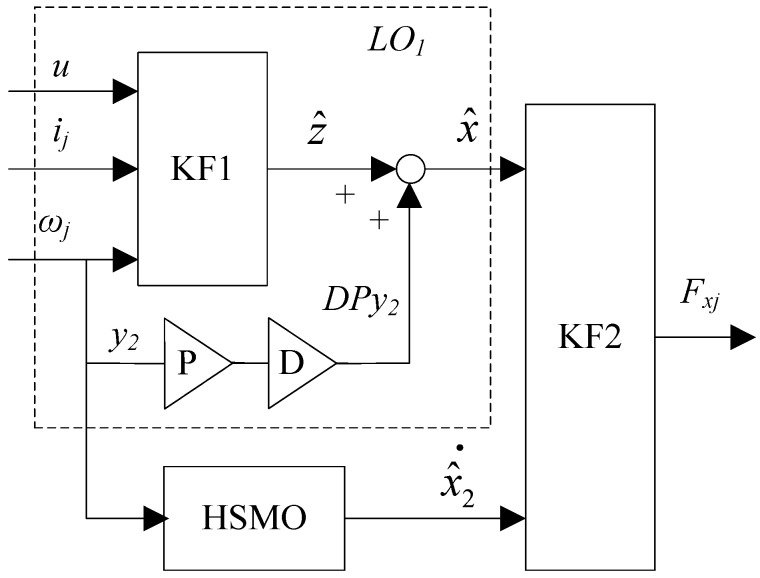
Diagram of the LFO.

The convergence of HSMO can be achieved by selecting suitable *k_x_*_2_ and *k_x_*_3_. The differential of ${\hat{x}}_{2}$ is estimated by the HSMO. According to (16), another KF is designed to estimate the longitudinal force, in which the $\hat{x}$ and ${\hat{x}}_{2}$ are seen as the known inputs and used as the pseudo measurements. The diagram of the LFO is displayed in Figure 3.

## 4. Sideslip Angle Estimation via the Fusion of Nonlinear Observers and Kalman Filter

Via Equations (1)–(3), the nonlinear state space equation of four-wheel vehicle model is expressed as:

(28) $$\left\{ \begin{array}{l} {\overset{\cdot }{x}}_{v}(t)=f_{v}(x_{v}(t),u_{v}(t))+w(t)\\ y_{v}(t)=h_{v}(x_{v}(t),u_{v}(t))+v(t) \end{array}\right.$$

where the input variables of state equation in Equation (28) consist of the velocity, the steering angle of front wheels, the longitudinal and lateral force of each wheel:

(29) $$\begin{array}{l} u_{v}={\left[\begin{array}{cc} v & \begin{array}{ccccccccc} \delta & F_{x1} & F_{x2} & F_{x3} & F_{x4} & F_{y1} & F_{y2} & F_{y3} & F_{y4} \end{array} \end{array}\right]}^{T}\\ ={\left[\begin{array}{cc} u_{v1} & \begin{array}{ccccccccc} u_{v2} & u_{v3} & u_{v4} & u_{v5} & u_{v6} & u_{v7} & u_{v8} & u_{v9} & u_{v10} \end{array} \end{array}\right]}^{T} \end{array}$$

The state variables are represented as:

(30) $$x_{v}(t)={\left[\begin{array}{cc} x_{v1} & \begin{array}{cc} x_{v2} & x_{v3} \end{array} \end{array}\right]}^{T}={\left[\begin{array}{cc} v_{yEKF} & \begin{array}{cc} \gamma_{EKF} & \beta_{EKF} \end{array} \end{array}\right]}^{T}$$

The measurement vectors are shown as:

(31) $$y_{v}={\left[\begin{array}{cc} y_{v1} & y_{v2} \end{array}\right]}^{T}$$

where *v_γM_* and *γ_M_* are the pseudo-measurements of vehicle lateral speed and yaw rate, achieved by the fusion of EKF (a posteriori estimation) and another specialised LO (transcendental estimation), and the fuzzy weight coefficient is designed to adjust the pseudo-measurements varying with driving manoeuvres and the degree of vehicle stability (the corresponding contents will be introduced in subsequent text). Therefore, the state equation of vehicle model is represented as:

(32) $$\left\{ \begin{array}{l} \overset{\cdot }{x_{v1}}=-x_{v2}\cdot u_{v1}+\frac{1}{m}[\left(u_{v3}+u_{v4}\right)\sin u_{v2}+(u_{v7}+u_{v8})\cos u_{v2}+u_{v9}+u_{v10}]\\ \overset{\cdot }{x_{v2}}=\frac{1}{I_{z}}[\left(u_{v3}+u_{v4}\right)l_{f}\sin u_{v2}-(u_{v9}+u_{v10})l_{r}+\left(u_{v7}+u_{v8}\right)l_{f}\cos u_{v2}\\ +(u_{v7}-u_{v8})b_{f}\sin u_{v2}-(u_{v3}-u_{v4})b_{f}\cos u_{v2}-(u_{v5}-u_{v6})b_{r}]\\ \overset{\cdot }{x_{v3}}=\frac{1}{mu_{v1}}[-(u_{v3}+u_{v4})\sin(x_{v3}-u_{v2})-(u_{v5}+u_{v6})\sin x_{v3}+\\ \left(u_{v7}+u_{v8}\right)\cos(x_{v3}-u_{v2})+(u_{v9}+u_{v10})\cos x_{v3}]-x_{v2} \end{array}\right.$$

The state-space Equations (1)–(3) are structured by the vehicle dynamics relationship, so the accuracy of (1)–(3) depends on the exact tire model. Therefore, we supplement the kinematics relationship into the measurement update process of EKF. Considering that the lateral vehicle speed is directly related to lateral acceleration, and the yaw rate is directly related to the longitudinal acceleration and the lateral acceleration, we utilize longitudinal acceleration and the lateral acceleration for the information compensation in measurement update, and the measurement equation of EKF can be expressed as:

(33) $$\left\{ \begin{array}{l} y_{v1}=v_{yM}+k_{11}a_{y}\\ y_{v2}=\gamma_{M}+k_{21}a_{x}+k_{22}a_{y} \end{array}\right.$$

where *a_x_* denotes the longitudinal acceleration, *a_y_* represents the lateral acceleration, *k*_11_, *k*_21_ and *k*_22_ are the appropriate compensation parameters, the transcendental estimations *v_yM_* and *γ_M_* are seen as the known inputs and pseudo-measurements in the EKF.

The extended Kalman filter (EKF) is a frequently-used algorithm for vehicle state estimation, which contains two parts: forecasting process and trimming process. In the forecasting process, the prediction of the next moment is obtained according to system state of the present moment. In the trimming process, the optimal estimation of the system is obtained by combining the observed results with the predicted values. With the discretization of state space Equation (28), the nonlinear stochastic state equation of the vehicle model is expressed as:

(34) $$\left\{ \begin{array}{l} x_{k+1}=f(x_{k},u_{k})+w_{k}\\ y_{k}=h(x_{k},u_{k})+v_{k} \end{array}\right.$$

The steps of EKF are given by:

(1) Forecasting process:

Calculate the forecast value:

(35) $${\overset{\wedge }{x}}_{k+1/k}={\overset{\wedge }{x}}_{k/k}+f({\overset{\wedge }{x}}_{k/k})T+A({\overset{\wedge }{x}}_{k/k})f({\overset{\wedge }{x}}_{k/k})T^{2}/2$$

Calculate the variance of prediction error:

(36) $$P_{k+1/k}=\varphi_{k}P_{k/k}\varphi_{k}^{T}+Q_{k}$$

where $A(x_{k/k})=\frac{\partial f(x)}{\partial x}|{}_{x={\overset{\wedge }{x}}_{k/k}}$,$\varphi_{k}=I+A({\overset{\wedge }{x}}_{k/k})T$.

(2) Trimming process:

Calculate the matrix of the Kalman gain:

(37) $$K_{k+1}=P_{k+1/k}H_{k+1}^{T}{\left(H_{k+1}P_{k+1/k}H_{k}^{T}+R_{k+1}\right)}^{-1}$$

Update the state estimation:

(38) $${\overset{\wedge }{x}}_{k+1/k+1}={\overset{\wedge }{x}}_{k+1/k}+K_{k+1}[y_{k+1}-h({\overset{\wedge }{x}}_{k+1/k})]$$

Update the error covariance:

(39) $$P_{k+1/k+1}=(I-K_{k+1}H_{k+1})P_{k+1/k}$$

where $H={\partial h(x)/\partial x|}_{x=\overset{\wedge }{x}(k/k)}$.

**Figure 4:**
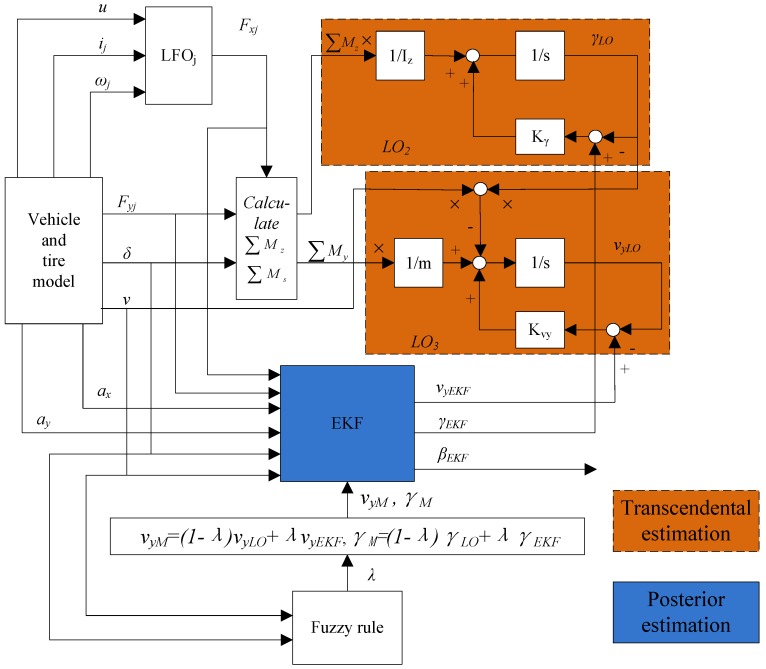
Architecture of the whole estimation method for vehicle state.

The diagram of the whole estimation system is shown is Figure 4, where based on Equation (2), the LO (marked as LO2) for the transcendental estimation of yaw rate is designed as:

(40) $${\overset{\cdot }{\gamma }}_{LO}=\frac{1}{I_{z}}\sum M_{z}+K_{\gamma }(\gamma_{EKF}-\gamma_{LO})$$

where the longitudinal forces of each wheel estimated by LFO, the lateral forces and the steering angle of front wheels are the inputs of LO2. The yaw rate estimation of LO2 is denoted by *γ_LO_*. According to Equation (1), another LO (marked as LO3) for the transcendental estimation of lateral vehicle speed is designed as:

(41) $${\overset{\cdot }{v}}_{yLO}=-\gamma \cdot v_{x}+\frac{1}{m}\sum M_{y}+K_{vy}(v_{yEKF}-v_{yLO})$$

where the longitudinal forces of each wheel estimated by LFO, the lateral forces, the steering angle of front wheels, the velocity heading of vehicle and the transcendental estimation of yaw rate by LO2 are the inputs of LO3. The lateral vehicle speed of LO3 is denoted by *v_yLO_*. Therefore, for LO2 and LO3, the estimation results of yaw rate and lateral vehicle speed by EKF are the pseudo- measurements.

In the design of LO2 and LO3, only the freedom of the yaw dynamics and lateral dynamics are considered, respectively. With the influences of noise and unknown interferes, this will lead to estimation errors and decrease of estimation accuracy owning to the integral accumulation, so the EKF is involved to the observer system for more accurate estimation results. The transcendental estimation *γ_LO_* and *v_yLO_* act as the pseudo measurements of EKF, and the posterior estimation *γ_EKF_* and *v_yEKF_* are convinced to be more exact than *γ_LO_* and *v_yLO_*.

**Figure 5:**
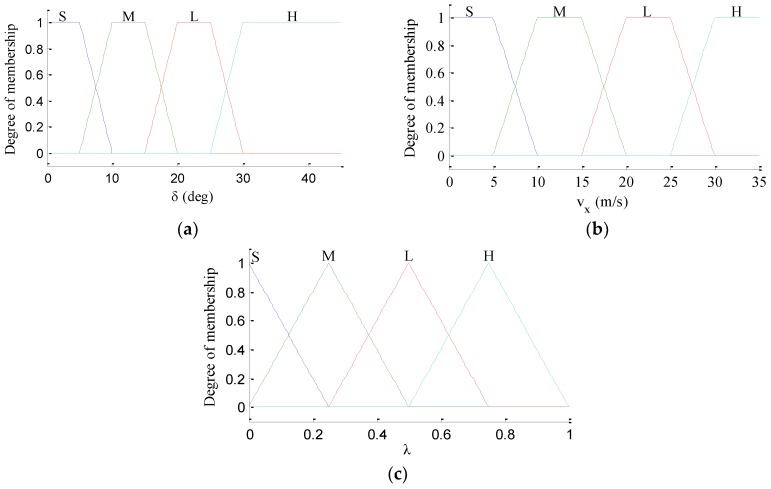
Membership functions of *v_x_*, *δ* and *λ*. (**a**) *v_x_*; (**b**) *δ*; (**c**) *λ*.

As we all know, along with the variation of vehicle driving manoeuvre and deterioration of vehicle stability degree, the estimation deviations will aggravate owing to the uncertainties of vehicle model such as nonlinear factors and unmodeled parameter variations. In order to weaken the influences caused by above factors, the fuzzy weight coefficients are respectively used to adaptively adjust the confidence level of *γ_LO_* and *v_yLO_* in the measurements of EKF. The fusion form is given by:

(42) $$\left\{ \begin{array}{l} v_{yM}=(1-\lambda )v_{yLo}+\lambda v_{yEKF}\\ \gamma_{M}=(1-\lambda )\gamma_{Lo}+\lambda \gamma_{EKF} \end{array}\right.$$

where *λ* is the fuzzy weight coefficient. The fuzzy controller in this paper is designed with two inputs, namely vehicle velocity *v_x_* and steering angle of front wheels *δ*, and one output namely fuzzy weight coefficient *λ*. The membership degree functions of *v_x_*, *δ* and *λ* are shown in Figure 5, in which the membership degree functions, classified as Small (S), Middle (M), Large (L) and Huge (H), are utilized to characterize the input and output variables. The fuzzy control rules for defuzzification are listed in Table 1.

To sum up, the whole hierarchical vehicle estimation method has two advantages. The first one is that the transcendental estimation is used as the pseudo-measurement, thus less sensors are required. The other one is that the information fusion between observers improves the estimation accuracy. The transcendental estimation and posterior estimation is individually utilized as the pseudo measurement of EKF and LO2/LO3. This design helps to suppress the estimation errors by the iteration and compensation of error between EKF and LO2/LO3.

## 5. Simulation Results

To evaluate the effectiveness of the designed LFO and proposed sideslip angle estimation method in this paper, the simulations are carried out in a high-fidelity CarSim-Simulink co-simulation platform. The parameters used in CarSim-Simulink joint simulation platform are the real vehicle parameters corresponding to the improved 4WID-EV in our laboratory and are obtained by measurement and identification. Relevant parameters of vehicle model, tire model and in-wheel motor model are listed in Table 2. In our simulation, CarSim is used to provide the whole vehicle model, and the estimation of longitudinal forces, lateral vehicle speed, yaw rate and sideslip angle are achieved in Matlab/Simulink.

### 5.1. Sine Steer Manoeuvre with a Constant Speed

**Figure 6:**
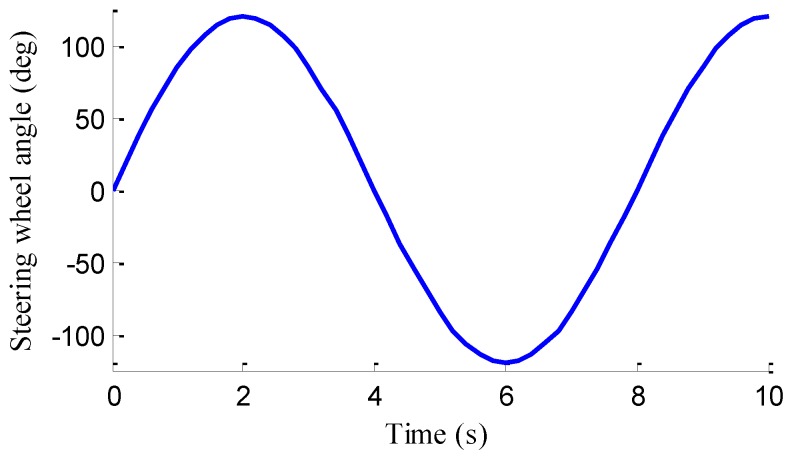
Steering wheel angle during the sine steer manoeuvre.

**Figure 7:**
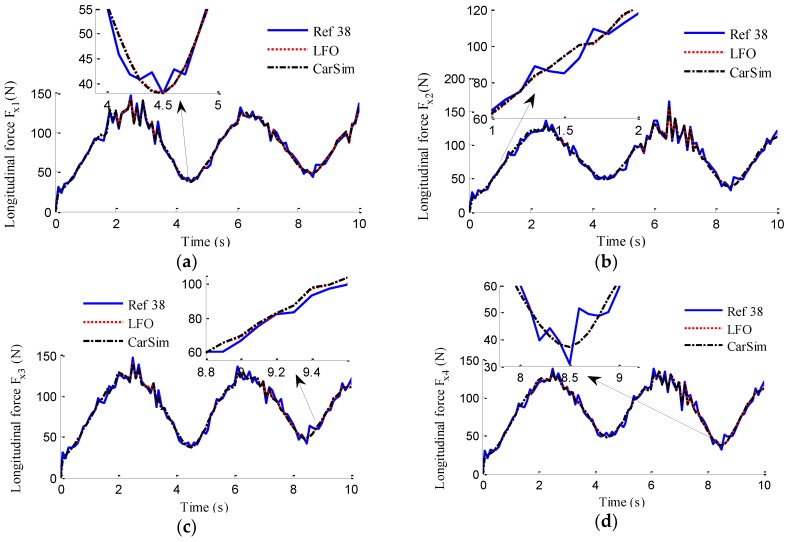
Estimation of longitudinal forces. (**a**) *F_x_*_1_; (**b**) *F_x_*_2_; (**c**) *F_x_*_3_; (**d**) *F_x_*_4_.

In the simulation, the road friction coefficient is set to be 0.6, the vehicle speed is maintained at a constant value of 60 km/h, and the steering wheel angle is a sine function with a magnitude of 120°, as shown in Figure 6. The designs of LFO for wheel 1, 2, 3, 4 are conducted, respectively. In previous works, no one has mentioned EDWM, and literature that specializes in longitudinal force estimation of in-wheel motor drive electric vehicle is still hard to find. Reference [38] proposed a longitudinal force estimation method by an unknown input observer. In order to compare with the proposed LFO, the unknown input observer (UIO) was introduced and used to estimate longitudinal force and denoted as [38] in the following figures. The estimation results of longitudinal forces in sine steer manoeuvres are shown in Figure 7. It can be found that the designed LFOs can estimate the longitudinal force accurately with the measurements of currents, speeds and voltages, that is to say, the estimated values of LFOs are reliable to replace the measurements of sensors as the inputs to LO2, LO3 and EKF.

**Figure 8:**
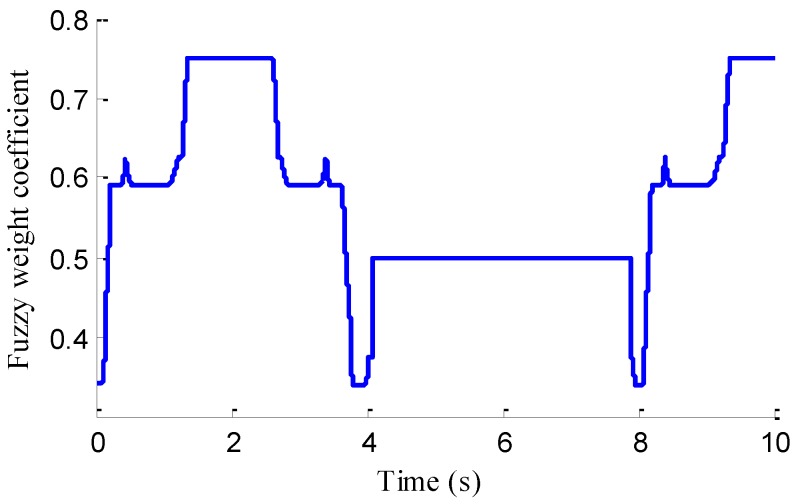
Fuzzy weight coefficient.

**Figure 9:**
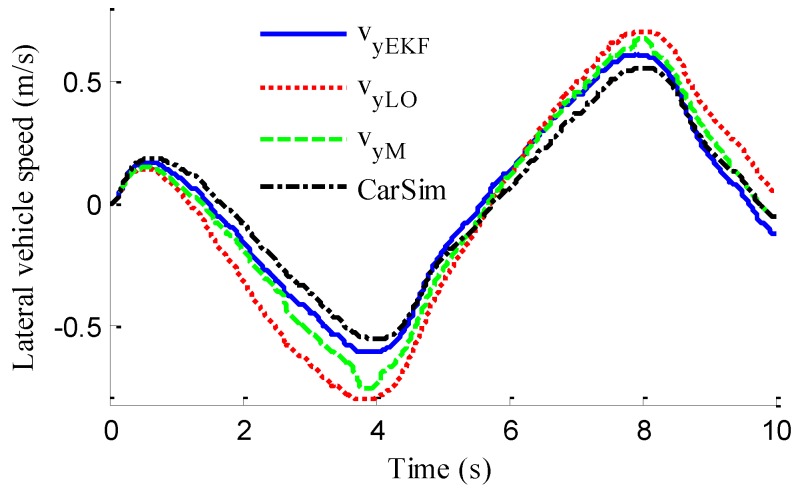
Estimation of lateral vehicle speed.

**Figure 10:**
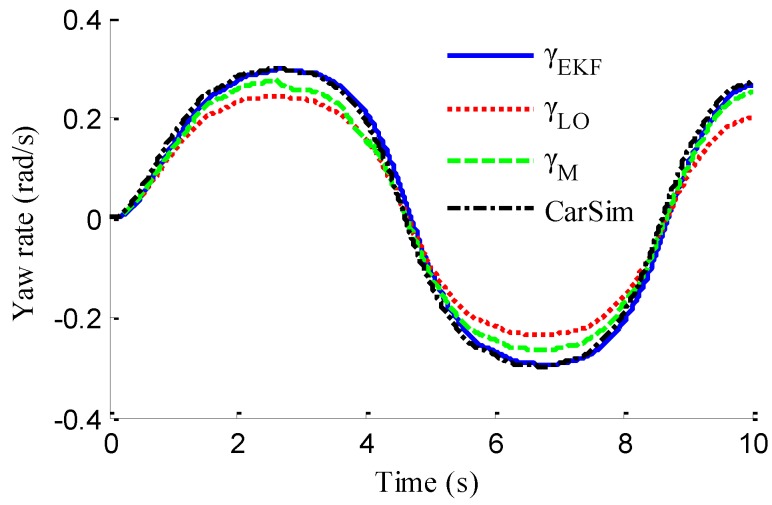
Estimation of yaw rate.

**Figure 11:**
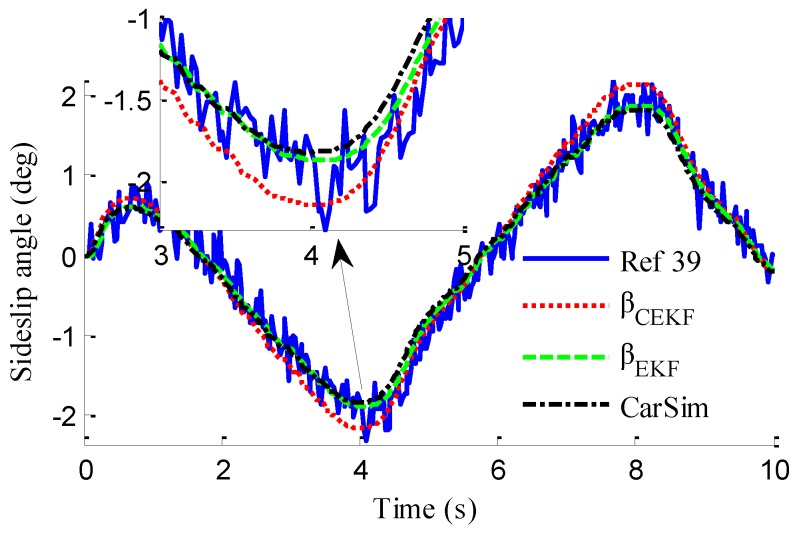
Estimation of sideslip angle.

In the verification process of the proposed vehicle state estimation strategy with observer iteration and information fusion, the commonly used EKF is applied to obtain the estimation. In the following text and figures, the estimation results using only EKF are denoted as CEKF. As shown in Figure 8, the fuzzy weight coefficient adaptively varies according to the variation of the sine steer manoeuvre, which indicates the fuzzy controller regulates the reliability level of the transcendental estimation with the change of steering wheel angle. Figure 9 shows the comparison of estimated values (such as *v_yEKF_*, *v_yLO_*, *v_yM_*,) and real values of lateral vehicle speed (from CarSim). There exists some error of the lateral vehicle speed estimation by LO3 when the vehicle running manoeuvres change rapidly. The update measurement *v_yM_* of EKF is revised by the adaptive fuzzy weight coefficient, so the accurate estimation of yaw rate is achieved by EKF. Figure 10 gives the comparison of the yaw rate estimation. The simulation results also prove the effect of fuzzy weight coefficient and the posterior estimation (*γ_EKF_*) is more accurate than transcendental estimation (*γ_LO_*). Reference [39] presented a novel sideslip angle estimation method, to further verify the performance of proposed sideslip angle estimation method in restraining noise and ensuring accuracy, the sideslip angle estimation method in reference [39] was used for comparison. As shown in Figure 11, the estimation of sideslip angle of proposed method can track the real sideslip angle in real-time, the estimation result with observer iteration and information fusion is better than the result with only EKF and the result of [39], in which the result with only EKF fails to track the real sideslip angle accurately and the result of [39] fails to remove noise interference. Therefore, the effectiveness of proposed estimation method is verified.

### 5.2. J-Turn Manoeuvre with a Varying Speed

**Figure 12:**
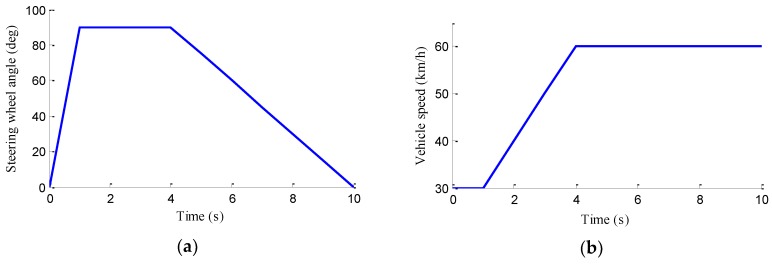
J-turn manoeuvre with a varying speed. (**a**) Steering wheel angle; (**b**) Vehicle speed.

In this case, the J-turn manoeuvre with the varying speed, as shown in Figure 12, is carried out. In simulation, the road friction coefficient is set to be 0.6. The vehicle speed is maintained at a constant 30 km/h in first second, after that, the vehicle accelerates to a speed of 60 km/h during 1 s to 4 s, and then remains at a constant speed. The steering wheel angle sharply increases to 90° during the first second and then is maintained during 1 s to 4 s, after that, it linearly decreases to 0° during the final 6 s.

**Figure 13:**
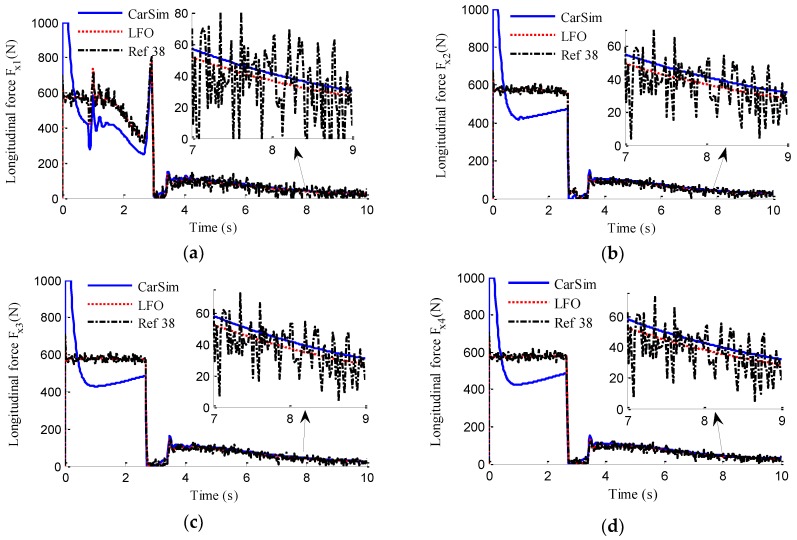
Estimation of longitudinal forces. (**a**) *F_x_*_1_; (**b**) *F_x_*_2_; (**c**) *F_x_*_3_; (**d**) *F_x_*_4_.

**Figure 14:**
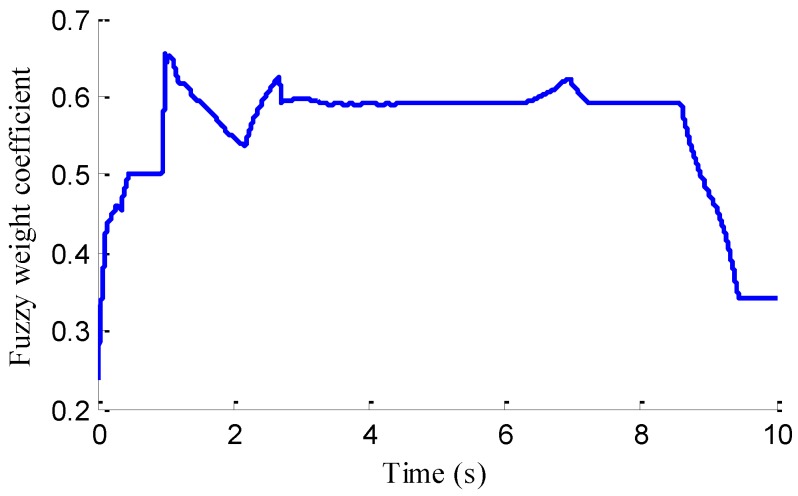
Fuzzy weight coefficient.

**Figure 15:**
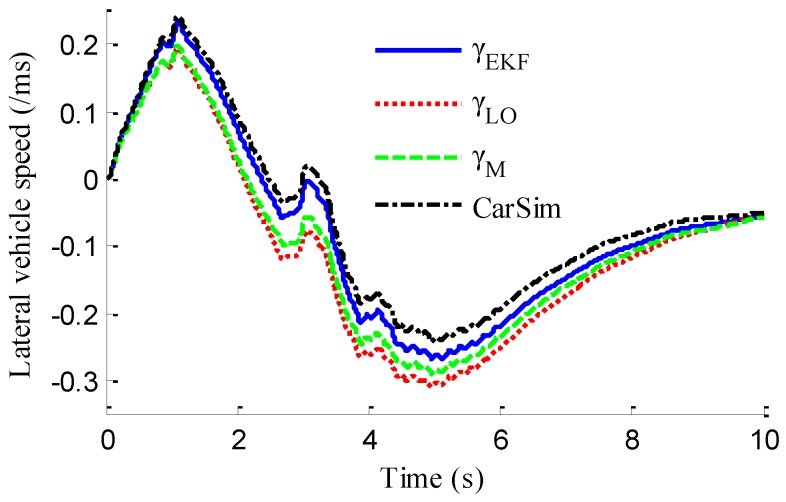
Estimation of lateral vehicle speed.

**Figure 16:**
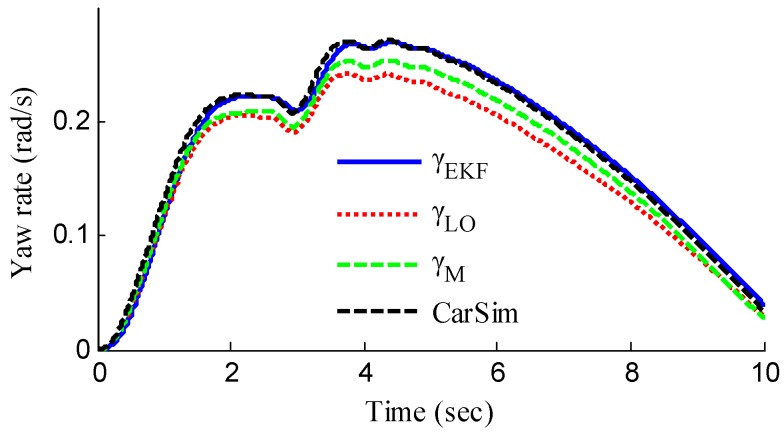
Estimation of yaw rate.

**Figure 17:**
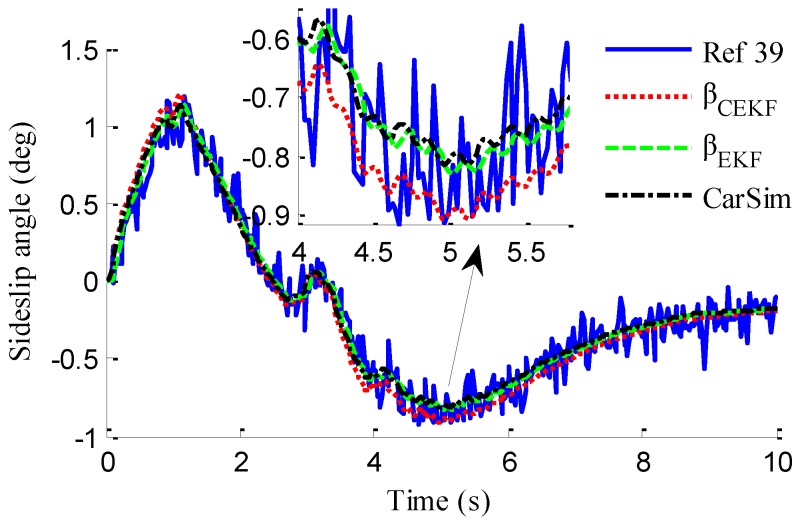
Estimation of sideslip angle.

Figure 13 represents the estimation results of longitudinal forces. It can be found that the vehicle actuating manoeuvre changes violently during 0 to 4 s, the estimation of longitudinal forces emerges some small fluctuations compared with the simulation results in Section 5.1, but the estimation error is relatively small and the estimated values approach stability after 4 s. This illustrates that the designed LFO has high estimation accuracy and anti-interference ability. Moreover, compared with common UIO, the presented LFO has better estimation accuracy and immunity to noise. As shown in Figure 14, like in Figure 8, the fuzzy weight coefficient adaptively varies according to the variation of driving manoeuvre to adjust the update-measurements of EKF. Figure 15 and Figure 16 respectively show the contrastive estimation of lateral vehicle speed and yaw rate. It can be found that the estimation performance via EKF is satisfactory even when the actuating manoeuvre of vehicle is complicated. Like the simulation sine steer manoeuvre results shown in Figure 17, the proposed estimation strategy can estimate the sideslip angle more precisely and has better estimation performance than that of only EKF and [39].

## 6. Experimental Results

In this section, the experimental verification is implemented for further validation about the performance of proposed estimation methodology. Considering that the sensors for immediate longitudinal force measurements are still unavailable on our four-wheel independent drive electric test vehicle, so the verification of LFO is carried out on the chassis dynamometer bench. The estimation of sideslip angle is testified by the road test results, in which the estimated longitudinal forces by LFOs are used as the credible pseudo-measurements.

### 6.1. Test on Chassis Dynamometer Bench

**Figure 18:**
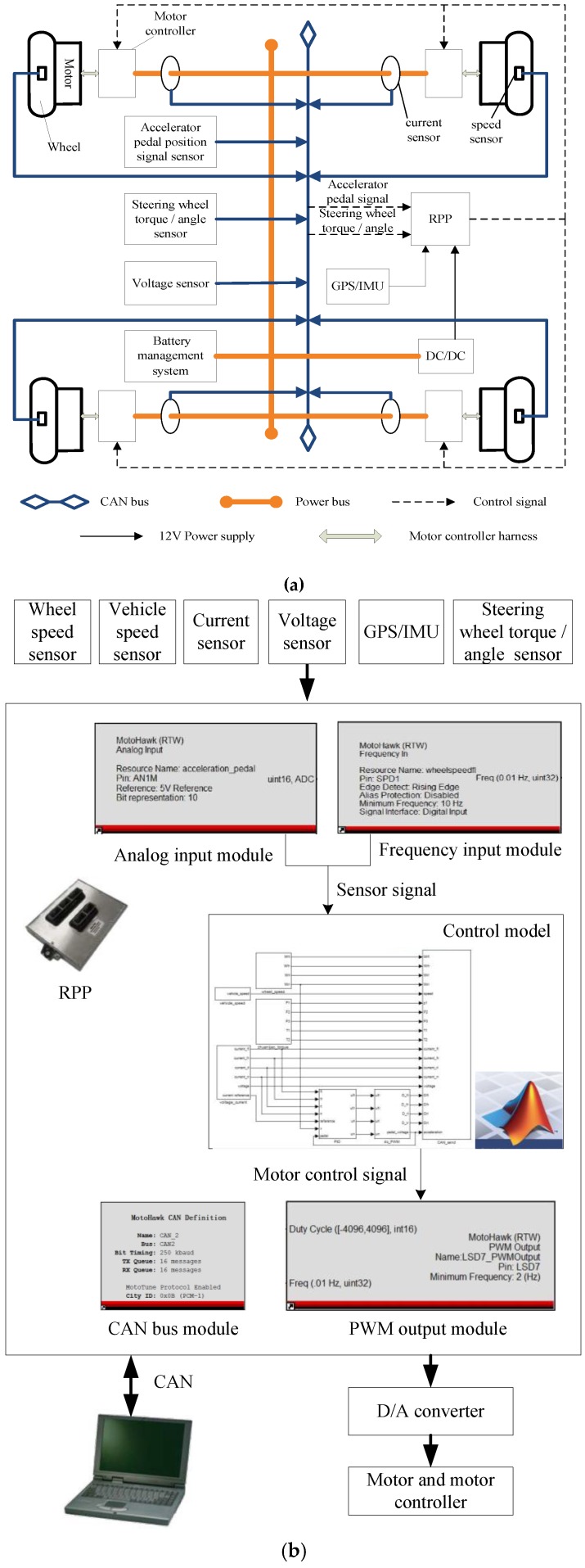

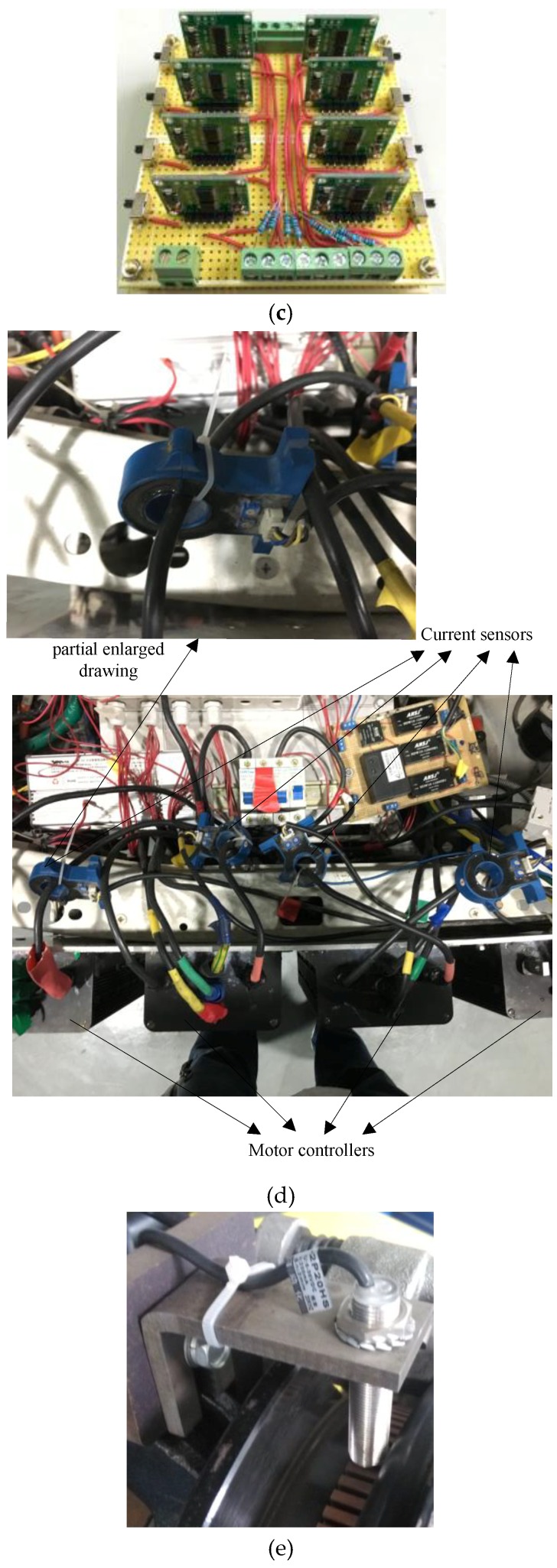
Control system and sensor network of 4WID-EV. (**a**) The electrical system of 4WID-EV; (**b**) Schematic diagram of sensor information collection; (**c**) D/A converter; (**d**) Current sensors; (**e**) Wheel speed sensors.

**Figure 19:**
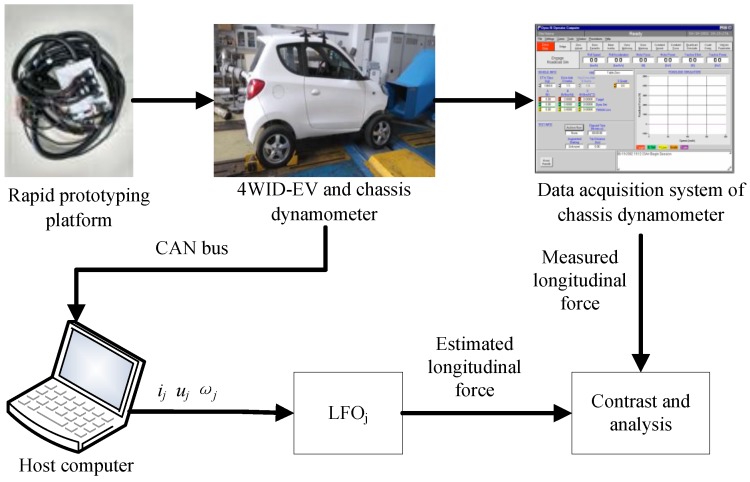
Vehicle test on chassis dynamometer bench.

The electrical system and sensor network of experimental vehicle are shown in Figure 18. The experimental vehicle is a four-wheel independent drive electric vehicle which is refitted from a single-motor drive electric vehicle and actuated by four in-wheel motors. The initial pure electric vehicle model of a ZHIDOU D1 produced by XDY Group (Linyi, China). The rated power, maximum torque and maximum speed of each in-wheel motor is 3 KW, 150 Nm and 750 r/min, respectively. The vehicle power source is a lithium iron phosphate battery pack with 72 V voltage output, and the vehicle control system is powered by 12 V power supply transformed by DC/DC converter. The whole vehicle control system is implemented via a rapid prototyping platform (RPP), which is an autonomous electronic control unit from development to production and produced by Eontronix Co., Ltd. (Beijing, China). The vehicle test on chassis dynamometer bench is shown in Figure 19. The vehicle sensor data are acquired in the form of analog quantity and frequency quantity. 

The real-time analysis and processing are used to solve the control signals for each in-wheel motor controller through the PWM output channel. D/A converter, as shown in Figure 18c, is used to achieve the new transformation of control signals compatible with motor controllers, so I/O matching between the motor controller and RPP is realized. The current sensor and wheel speed sensor are shown in Figure 18d,e. The voltage information is provided by the battery management system of original electric vehicle and can be obtained directly by CAN bus. The software MotoHawk-CAN Training is a rapid controls system development tool that allows controls engineers to quickly create controls software within Simulink diagrams, so in MotoHawk, we can define the input and output module to acquire the sensor information and output the control signals. Moreover, the integrated CAN Message Read module can be invoked directly with the INS CAN attitude are defined. Currents, speeds and voltages of in-wheel motors were measured with corresponding sensors and recorded by the host computer through CAN bus using CAN tools of Vehicle SPY 3. The Vehicle Spy was produced by Intrepid Control Systems, Inc. China (Shanghai, China). The longitudinal forces were obtained by the data acquisition system of a chassis dynamometer.

**Figure 20:**
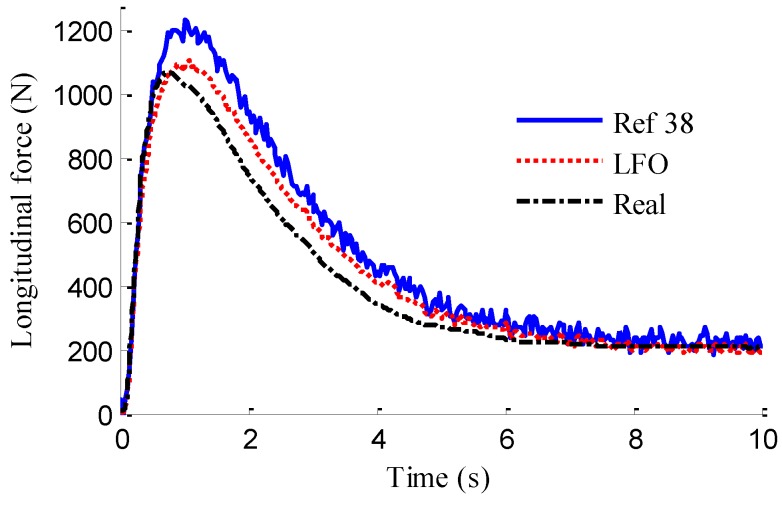
Estimation of longitudinal force.

In this paper, we choose the experimental data of one electric driving wheel (front-right wheel) for the validation of proposed LFO, the experimental results are shown in Figure 20. It can be found that there exists some estimation error in the acceleration process, but the error is finite. The LFO can track the longitudinal force with high accuracy after 5 s when the driving condition approaches to the manoeuvre of uniform speed and the global estimation accuracy of LFO is obviously better than common UIO.

### 6.2. Road Test

**Figure 21:**
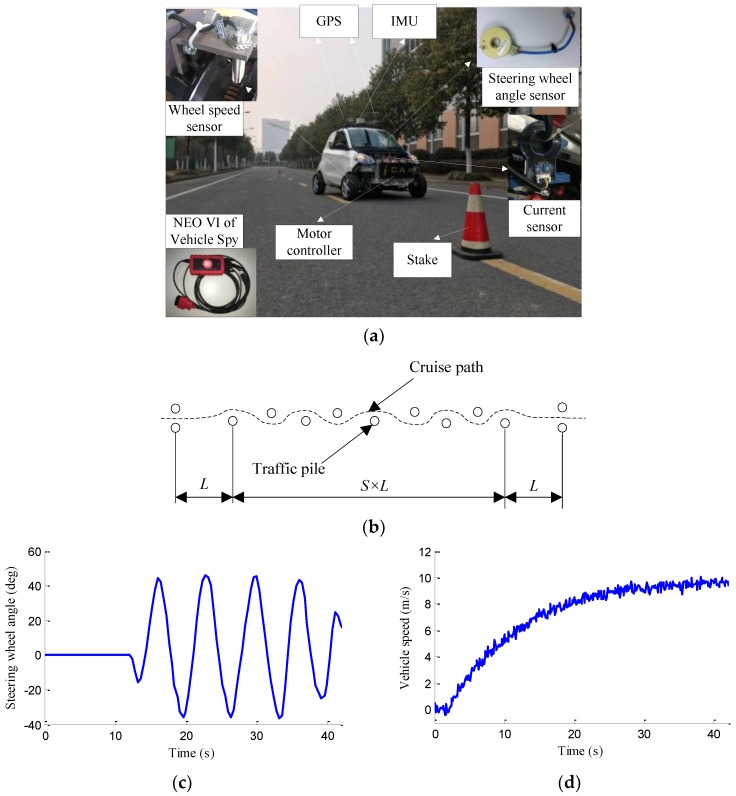
Road test. (**a**) 4WID-EV and experimental scene; (**b**) Vehicle trajectory; (**c**) Steering wheel angle; (**d**) Vehicle speed.

As shown in Figure 21, the road test is implemented on a flat asphalt road, 10 traffic piles are placed on the road, and the distance between every two adjacent piles is 30 m. The lateral vehicle speed, yaw rate and sideslip angle are measured by the high-precision difference global position system (GPS) and inertial measurement unit (IMU), which was produced by Crossbow Technology, Inc. (Beijing, China). The vehicle speed cruising is regulated well by a speed controller, as shown in Figure 21d, where the vehicle speed is basically stable at around 10 m/s after 20 s of vehicle acceleration.

**Figure 22:**
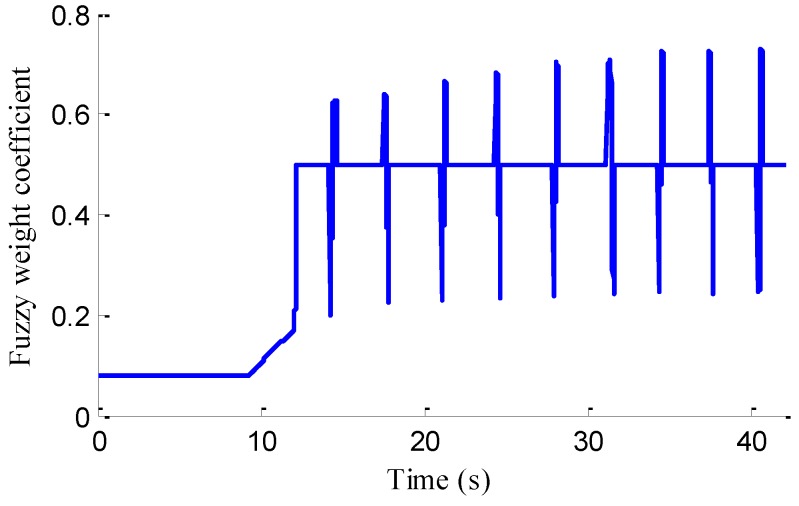
Fuzzy weight coefficient.

**Figure 23:**
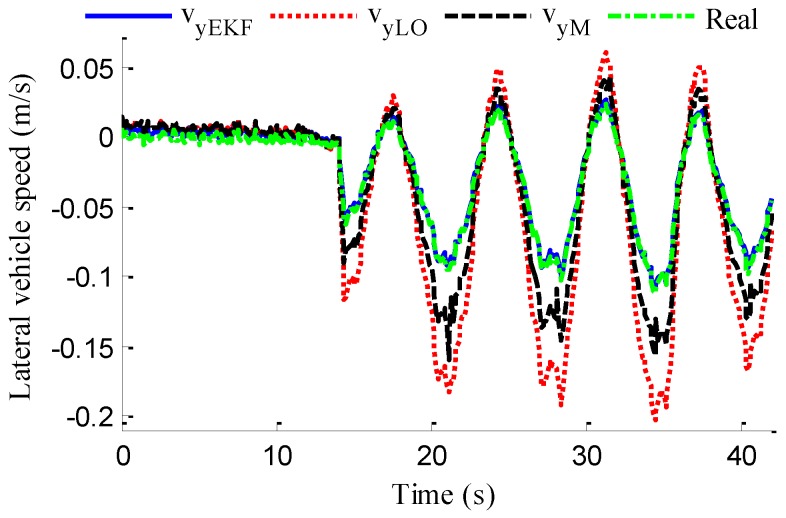
Estimation of lateral vehicle speed.

**Figure 24:**
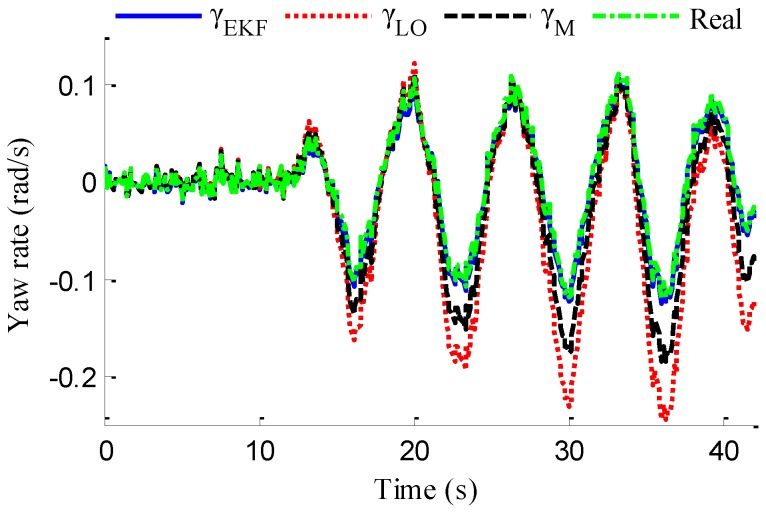
Estimation of yaw rate.

**Figure 25:**
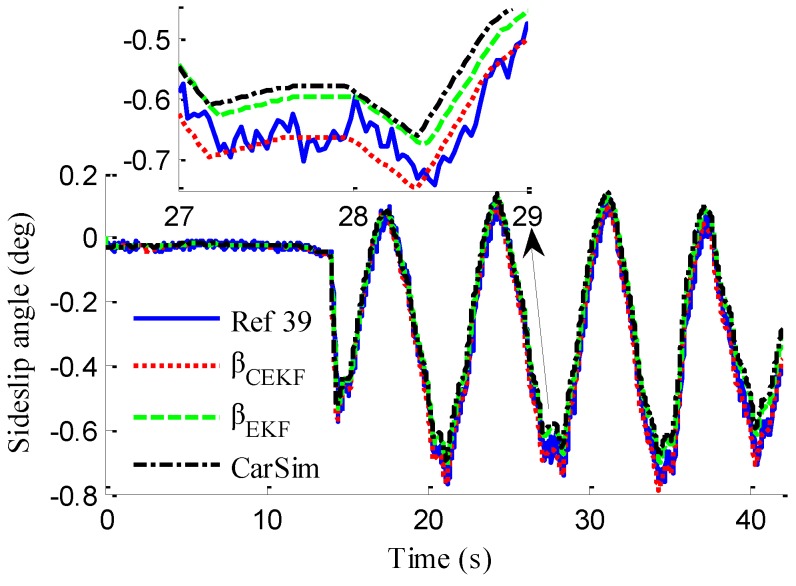
Estimation of sideslip angle.

As shown in Figure 22, same as the simulation results, the fuzzy weight coefficient adaptively varies with the variation of driving manoeuvres. As the vehicle speed is relatively low, the fuzzy weight coefficient is mainly affected by the change of steering wheel angle. Figure 23 and Figure 24 show the comparison between estimated values and real values of lateral vehicle speed and yaw rate, respectively. One can see that the proposed estimation method with observer fusion improves the estimation accuracy of the posterior estimation and the application of EKF can effectively suppress the influences of noise. Figure 25 shows the performance of the proposed method in the estimation of sideslip angle, it can be found that the performance of proposed estimation strategy is satisfactory, and the result of only EKF can track the sideslip angel generally, but the estimation accuracy is lower, while the method of [39] can track the sideslip angel in the overall trend, but the estimation result is significantly affected by noise. 

For further validation, the root-mean-square (RMS) error *E_RMS_* between measurement and estimation is used for quantitative evaluation and can be computed by the following equation:

(43) $$E_{RMS}=\sqrt{\frac{1}{N_{s}}\sum_{i=1}^{N_{s}}{\left({\hat{x}}_{i}-x_{i}\right)}^{2}}$$

where *N_s_* is the number of samples, $x_{i}$ and ${\hat{x}}_{i}$ denote the measured vehicle state and estimated vehicle state at the *i*th sample. Through computation, the *E_RMS_* of *v_yEKF_*, *v_yLO_*, and *v_yM_* is 0.0418, 0.1174 and 0.1851, respectively, the *E_RMS_* of *γ_yEKF_*, *γ_LO_*, and *γ_M_* is 0.1032, 0.1493 and 0.1967, respectively, and the *E_RMS_* of *β_CEKF_*, [39], and *β_EKF_* is 0.1438, 0.0703 and 0.0657, respectively. It can be seen that the *E_RMS_* of EKF estimation with iteration and fusion is much smaller than that of LO2, LO3, only EKF, and [39]. This indicates that the method of observer iteration and information fusion enhances the accuracy and reliability of sideslip angle estimation. The feasibility in practice of the proposed estimation method in this paper is thus verified.

## 7. Conclusions

In this work, we proposed a novel estimation method of longitudinal force and sideslip angle for 4WID-EVs. Considering that the longitudinal force is the unknown input of EDWM, we decoupled the system by model reduction and achieved the reconstruction equation of longitudinal force. The Luenberger observer and high-order sliding mode observer were designed to estimate the longitudinal force, and the Kalman filter was used to improve the accuracy of longitudinal force estimation. On the basis of LFO design, we proposed a sideslip angle estimation method with observer iteration and information fusion, in which the Luenberger observer was used to get a transcendental estimation and the EKF was used to get an a posteriori estimation, the estimations of Luenberger observer were regarded as the inputs of EKF from pseudo-sensors. The fuzzy controller was devised to adjust the confidence level of the LFO estimation and EKF estimation. The simulations of sine steer manoeuvres with constant speed and J-turn manoeuvres with varying speed were carried out, and the results show that the presented LFO can estimate the longitudinal force precisely, and the observer iteration-based sideslip angle estimation strategy improves the accuracy and reliability of sideslip angle estimation, the designed fuzzy controller can adaptively regulate the fuzzy weight with the variations of vehicle manoeuvre. For further validation, the test on chassis dynamometer bench and road test was implemented, and the performance of proposed estimation method was verified.

## Figures and Tables

**Table 1:** Fuzzy rules.

*v_x_*	*δ*			
	S	M	L	H
S	S	M	M	L
M	M	L	L	H
L	L	H	H	H
H	L	H	H	H

**Table 2:** Parameters of the vehicle, tires and in-wheel motor.

Symbol	Parameters	Value and Units
*m*	Vehicle mass	710 kg
*r*	Effective radius of wheel	0.245 m
*l_f_*	Distances from vehicle gravity center to the front axle	0.795 m
*l_r_*	Distances from vehicle gravity center to the rear axle	0.975 m
*b_f_*, *b_r_*	Half treads of the front(rear) wheels	0.775 m
*C_f_*	Equivalent cornering stiffness of front wheel	60,000 N/rad
*C_r_*	Equivalent cornering stiffness of rear wheel	40,000 N/rad
*I_z_*	Moment of inertia	1000 kg·m^2^
*R*	Equivalent resistance of winding	0.688 Ω
*K_a_*	Inverse electromotive force coefficient	0.06 Nm/A
*K_t_*	Motor torque constant	11.43 Nm/A
*J*	Sum of inertia moment of wheel and motor	7.143 kg·m^2^
*b*	Damping coefficient	0.643 Nm·s/rad
*L*	Equivalent inductance of winding	0.125 H
